# HPV-positive oropharyngeal squamous cell carcinoma is associated with *TIMP3* and *CADM1* promoter hypermethylation

**DOI:** 10.1002/cam4.313

**Published:** 2014-07-26

**Authors:** Pauline M W van Kempen, Liselotte van Bockel, Weibel W Braunius, Cathy B Moelans, Marina van Olst, Rick de Jong, Inge Stegeman, Paul J van Diest, Wilko Grolman, Stefan M Willems

**Affiliations:** 1 Department of Otorhinolaryngology – Head and Neck Surgery, University Medical Center UtrechtUtrecht, The Netherlands; 2 Brain Center Rudolf Magnus, University Medical Center UtrechtUtrecht, The Netherlands; 3 Department of Radiotherapy, University Medical Center UtrechtUtrecht, The Netherlands; 4 Department of Pathology, University Medical Center UtrechtUtrecht, The Netherlands; 5 Department of Molecular Carcinogenesis, Netherlands Cancer InstitutePlesmanlaan 121, Amsterdam, The Netherlands

**Keywords:** CADM1, CHFR, HPV, hypermethylation, oropharynx squamous cell carcinoma, TIMP3

## Abstract

Oropharyngeal squamous cell carcinoma (OPSCC) is associated with human papillomavirus (HPV) in a proportion of tumors. HPV-positive OPSCC is considered a distinct molecular entity with a prognostic advantage compared to HPV-negative cases. Silencing of cancer-related genes by DNA promoter hypermethylation may play an important role in the development of OPSCC. Hence, we examined promoter methylation status in 24 common tumor suppressor genes in a group of 200 OPSCCs to determine differentially methylated genes in HPV-positive versus HPV-negative primary OPSCC. Methylation status was correlated with HPV status, clinical features, and patient survival using multivariate methods. Additionally, methylation status of 16 cervical squamous cell carcinomas (SCC) was compared with HPV-positive OPSCC. Using methylation-specific probe amplification, HPV-positive OPSCC showed a significantly higher cumulative methylation index (CMI) compared to HPV-negative OPSCC (*P*=0.008). For the genes *CDH13*, *DAPK1*, and *RARB*, both HPV-positive and HPV-negative OPSCC showed promoter hypermethylation in at least 20% of the tumors. HPV status was found to be an independent predictor of promoter hypermethylation of *CADM1* (*P* < 0.001), *CHFR* (*P* = 0.027), and *TIMP3* (*P* < 0.001). *CADM1* and *CHFR* showed similar methylation patterns in OPSCC and cervical SCC, but TIMP3 showed no methylation in cervical SCC in contrast to OPSCC. Methylation status of neither individual gene nor CMI was associated with survival. These results suggest that HPV-positive tumors are to a greater extent driven by promotor hypermethylation in these tumor suppressor genes. Especially *CADM1* and *TIMP3* are significantly more frequently hypermethylated in HPV-positive OPSCC and *CHFR* in HPV-negative tumors.

## Introduction

Head and neck squamous cell carcinoma (HNSCC) is the sixth most common cancer worldwide 1. Most cases are diagnosed in a late stage and therefore the 5-year overall survival is still relatively poor at ∼50% 2 despite recent improvements in treatment and detection methods 3.

Besides known risk factors such as alcohol consumption and tobacco use, human papillomavirus (HPV, especially type 16) has been identified as an independent risk factor for a subset of HNSCC, in particular OPSCC 1,4,5. Patients with OPSCC testing positive for HPV generally respond more favorable to chemotherapy and radiation than patients with a negative HPV status. Currently, HPV-positive OPSCC is considered a distinct molecular and clinical entity compared to HPV-negative OPSCC 6,7. Therefore, identification of HPV status can serve as a biomarker for survival and may play an important role in choice of treatment in near future. However, the exact mechanism underlying this difference in clinical behavior between HPV-positive and HPV-negative OPSCC remains poorly understood.

Epigenetic changes are thought to be an early event in the carcinogenesis in various human cancers and could (at least) partly be responsible for the difference between HPV-positive and HPV-negative in molecular and clinical behavior 8,9. Hypermethylation in promoter regions of tumor suppressor genes is the best characterized epigenetic change and leads to transcriptional silencing 10,11. DNA methylation is reversible and therefore a potential target for therapy and can serve as biomarker for therapy response and prognosis 12,13. Several genes have already been identified as aberrantly methylated between HPV-positive and HPV-negative tumors 14–21. These studies suggest that HPV-positive tumors are more driven by methylation alterations in the promoter region and HPV-negative tumors by global hypomethylation of the genome 22,23. However, most of these studies evaluated a single or limited number of candidate genes in HNSCC as a whole, and most of them did not specifically focus on OPSCC. This is important because carcinogenesis of HNSCC shows site-specific features and HPV positivity is particularly associated with the oropharynx. More insight into the underlying molecular mechanism of HPV-positive OPSCC and the better prognosis of these tumors will contribute to novel biomarkers and targets for individualized cancer treatment of HPV-positive and HPV-negative tumors. Our aim was therefore to investigate the role of promoter hypermethylation of 24 tumor suppressor genes in HPV-positive and HPV-negative OPSCC using methylation-specific multiplex ligation-dependent probe amplification (MS-MLPA). Additionally, we correlated methylation patterns with clinical features and prognosis. Finally, the results of HPV-positive OPSCCs were compared to a group of cervical SCC.

## Materials and Methods

### Patient selection

We selected all patients who were diagnosed with a first primary OPSCC at the University Medical Center Utrecht between August 1997 and December 2011. Exclusion criteria were a previous history of HNSCC, histologic abnormalities including inflammation, and dysplastic lesions, leaving 210 patients. Pathological, demographical, clinical, and survival data were retrieved from electronic medical records. Since we used leftover material, no ethical approval is required according to Dutch national ethical guidelines. Anonymous or coded use of leftover tissue for scientific purposes is part of standard treatment agreement with patients in our center 24. All this information was handled in a coded fashion, according to Dutch national ethical guidelines (Code for proper secondary use of human tissue, Dutch Federation of Medical Scientific Societies). HPV status was determined for all tumors by a combination of p16 immunohistochemistry (IHC) and linear array as described further. In addition, normal oropharynx formalin-fixed paraffin embedded (FFPE) tissue of 10 patients having an unknown primary tumor in head and neck region was taken along as control for OPSCC experiments and 10 normal cervix tissues for the evaluation of methylation in cervix SCC. These patients were comparable in age with the patients in our study cohort.

### DNA extraction

Tumor and normal areas were identified on HE slides by a dedicated head and neck pathologist (S. M. W.) and corresponding areas were dissected from 5 *μ*m paraffin blank slides using a scalpel. Tumor cell percentage was at least 30%. DNA isolation was achieved by suspending in direct lysis buffer (50 mmol/L Tris-HCL, pH 8.0; 0.5% Tween 20) and overnight incubation in proteinase K (10 mg/mL; Roche, Almere, the Netherlands) at 56°C. Proteinase K was then inactivated by heating the lysate to 100°C for 10 min. For further analysis the supernatant was used. DNA content was measured with a spectrophotometer. The DNA concentration of the samples was varying between 43.2, and 1901.9 ng/*μ*L. The resulting DNA was stored at −20°C until use.

### HPV DNA detection

Human papillomavirus type 16 (HPV-16) tumors were determined according to the algorithm of Smeets et al. 25. First, a slide of FFPE tumor was incubated for 1 h at room temperature with an antibody against p16 (clone 16P07; Neomarkers, Fremont, CA). All primary antibodies were diluted in phosphate-buffered saline (PBS), 0.1% sodium azide, and 1% bovine serum albumin. The signal was amplified using Brightvision poly-HRP anti-mouse, -rabbit, -rat (DPVO-HRP; Immunologic, Duiven, the Netherlands) and developed with diaminobenzidine plus for 10 min, followed by counterstaining with haematoxylin, dehydration in alcohol, and mounting. The positive control was HPV16-positive tonsil tissue and the negative control was normal skin tissue. A case was considered p16-positive when at least 70% of neoplastic cells showed strong (2+/3+) nuclear and/or cytoplasmatic staining. In case of positive p16 staining, a linear array analysis for confirmation was followed. PCR was performed using the Linear array HPV Genotyping test (S01710; Roche) as well as the Linear array Detection kit (S03373; Roche) according to the manufacturer's protocols. The master mix contains primers for the amplification of the L1 region of more than 30 genotypes.

### MS-MLPA analysis

For methylation analysis in tumor and control tissue, MS-MLPA (MRC Holland, Amsterdam, the Netherlands) was performed according to the manufacturer's instructions for a set of 24 tumor suppressor genes (Probe mix ME001-C2; MRC Holland, Amsterdam, The Netherlands). The choice for probe mix ME001-C2 was based on a thorough literature search indicating that genes specific for this particular tumor suppressor kit showed frequent methylation in head and neck cancer in association with prognosis and that hypermethylation of some promoter regions were associated with HPV in cancer of the cervix 26,27. A short overview of the functions of these genes is presented in Table S1.

The principle of MS-MLPA has been described elsewhere in more detail 28. In short, MS-MLPA kits contain probes with a specific restriction site for the methylation-sensitive enzyme, Hha1. After denaturation of isolated DNA, the probe mix was added and the samples were incubated overnight at 60°C. Each sample was divided into two tubes, one of which incubated with ligase buffer as a standard MLPA reaction and the other with both ligase buffer and with Hha1. Next, primers and PCR buffer were added and a PCR was performed. In the tube with Hha1 enzyme, methylated DNA is prevented from being digested by the methylation-sensitive restriction enzyme and therefore the target region is ligated and amplified by PCR. Unmethylated DNA is digested and not exponentially amplified by PCR. All runs were performed on a Veriti 96-well thermal cycler (Applied Biosystems, Foster City, CA). The first 20 cases were run in duplicate, but because of comparable results of duplicates all other cases were run in singular. SssI methylated cell line DNA was used as positive (100% methylated) control and DNA derived from human blood from a healthy volunteers as a negative (unmethylated) control. Both were taken along in each MS-MLPA run in duplicate. Samples with a DNA concentration above 700 ng/*μ*L were diluted 1:1 in Milli-Q water (Merck Millipore, MRAPP092) and above 1500 ng/*μ*L were diluted 1:2 in Milli-Q water (Merck Millipore).

Reaction products were separated by electrophoresis on an ABI 3730 capillary sequencer (Applied Biosystems). Methylation status analysis was performed with Genemapper software v4.1 (Applied Biosystems) and Coffalyser.NET analysis (MRC Holland) software. First intrasample normalization was carried out by comparing the signal of each probe by the signal of 15 internal reference probes in the MS-MLPA kit. The methylation percentage was then calculated for each probe as the ratio between normalized probe peaks from the undigested sample and the corresponding HhaI digested sample. For the genes, *MLH1* and *RASSF1A*, two probes for different CpG islands were available. Mean value of two probes for same gene were used for analysis. Promoter methylation analysis in normal oropharynx and cervix (control) tissue was performed using the same method and tumor suppressor kit.

According to previous cell line experiments and previous experiences, the cutoff level for promoter hypermethylation was set at 15% 29–31. The cumulative methylation index (CMI) was calculated as the sum of the methylation percentages of the individual genes, as before 32.

### Comparison to squamous cell carcinoma of the cervix

Development of cancer of the cervix is causally related to infection with high-risk human papillomavirus, mainly types 16 and 18 33. To study whether differences in promoter hypermethylation might be HPV-related events, we compared results in HPV-positive OPSCCs with HPV-positive (mainly HPV type 16) cases of cervical SCC. HPV status was examined using the same algorithm as for OPSCC and the same tumor suppressor kit (Probe mix ME001-C2) was used for methylation analysis.

### Tissue microarrays and immunohistochemical staining

Hematoxylin and eosin (H&E)-stained sections were cut from each donor block and examined by an experienced head and neck pathologist (S. M. W.) to mark representative tumor regions. A fully automated tissue microarray instrument (Beecher Instruments) was used to acquire tissue cores from the donor block from our patient's cohort. From each block, three cores with a diameter of 0.6 mm were subsequently placed in an empty paraffin block. Tissue microarrays included 193 of 210 patients from our cohort. In addition, normal oropharynx (*n* = 10) was added as control. The tissue microarray is cut in 4-*μ*m paraffin sections using standard techniques. Immunohistochemistry was performed using a monoclonal-rabbit *CHFR* antibody (Clone, D40H6; Cell Signaling Technology, Danvers, MA) in a 1:300 dilution and *CADM1* antibody (Atlas antibodies, Sigma-Aldrich, St Louis, MO; S4945) in a 1:10,000 dilution. For all stainings, slides were deparaffinized in xylene and rehydrated in decreasing ethanol dilutions. Endogenous peroxidase was blocked with H_2_O_2_ in PBS followed by antigen retrieval by boiling in an EDTA buffer, pH = 9. After a cooling down period of 30 min in the buffers, the tissue slides were incubated with the primary antibodies for 60 min at room temperature. For detection of the primary antibodies the tissue slides were incubated with poly-HRP goat/rabbit/rat (Ready to use; Brightvision, immunologic Duiven, the Netherlands). Between these steps, slides were washed with PBS. Finally, peroxidase activity was developed with diaminobenzidine for 10 min, slides were counterstained with hematoxylin, and dehydrated in increasing alcohol dilutions. Normal lung tissue was taken along as positive control.

Scoring of IHC slides was performed by two independent observers (S. M. W. and P. M. W.), blinded to the clinical characteristics. A core was considered lost or inadequate if it contained less than 5% tumor tissue or when more than 95% of the core contained no tissue anymore. *CHFR* was semiquantitatively scored as previously described by Pillai et al. 34. *CADM1* was scored for cytoplasmatic staining according to the scoring method described by Botling et al. 35.

### Statistics

The Mann–Whitney test was used to compare CMI between HPV-positive and HPV-negative cases and to calculate differences in CMI between HPV-positive OPSCC and cervical cases. Pearson *χ*^2^ test (or Fisher's exact when appropriate) for categorical variables and ANOVA for continuous variables were used to compare the frequency of methylation for individual genes between HPV-positive and HPV-negative tumors. The same tests were used to compare frequency of methylation between cervical cancer and HPV-positive OPSCC. Correction for multiple comparisons was applied by resetting the 0.05 threshold to 0.05/24 = 0.002 (Bonferroni correction). The following clinicopathological features were dichotomized: tumor size (T1/2 vs. T3/4), stage (I/II vs. III/IV), nodal stage (0 vs. 1–3). Backward logistic regression was performed to compare methylation in HPV-positive and HPV-negative tumors and to compare HPV-positive and cervical cancer, taking significant differences between the two groups into account. Odds ratios (OR) and 95% confidence intervals (CI) were calculated. Patient survival was examined first using Kaplan–Meier survival curves, and differences between strata were tested using log-rank test. To adjust for additional variables related to patient survival, Cox regression analysis was used. Next, we explored the association between methylation status of *CADM1* and *CHFR* and protein expression using the nonparametric Spearman's correlation coefficient between the mean protein expression levels of *CADM1* or *CHFR* and methylation percentage as continuous variable. Next, we calculated the association between promoter hypermethylation dichotomized (cutoff value 15%) of *CHFR* and *CADM1* and protein expression of these proteins dichotomized (high vs. low) using Pearson *χ*^2^ test (or Fisher's exact when appropriate). Finally, we evaluated the relation between HPV status and *CHFR* or *CADM1* expression (low vs. high) using Pearson *χ*^2^ test (or Fisher's exact when appropriate). All statistical analyses were performed using IBM SPSS 20.0 statistical software (Chicago, IL).

## Results

### Patient characteristics

The original group of OPSCCs consisted of 210 patients, however, in 10 cases the amount of DNA was insufficient, leaving 200 cases for further analyses. Of 200 cases stained for p16, 69 were scored as positive. Of 69 p16-positive tumors 43 were confirmed to be HPV-positive by PCR, of which 42 were high-risk HPV. All of the 42 high-risk HPV-positive were HPV16 and two were coinfected with HPV33 or HPV52, resulting in a HPV prevalence of 21%. HPV16 was detected most frequently in base of tongue, tonsillar fossa, and tonsil. Distribution of relevant clinical features between HPV-positive and HPV-negative OPSCCs is shown in Table 1. In addressing patients' characteristics, HPV-negative patients were significantly more frequent smokers and excessive alcohol users. HPV-positive patients had clinically lower T stage and higher N stage at the time of diagnosis compared to HPV-negative cases. No significant differences were found in the remaining features, including age, gender, and treatment. All 10 normal oropharynx tissues taken as control had an equal distribution in age and gender compared to tumor tissue.

**Table 1:** Characteristics of 200 patients by HPV status

Patient or tumor characteristics	HPV-positive (%)	HPV-negative (%)	*P*-value	Control (%)
No. of cases	42 (21)	158 (79)	–	10
Age				
Average (range)	58.2 (35–80)	59.8 (40–88)	0.341	59.2 (27–93)
Sex				
Male	33 (79)	106 (67)	0.151	6 (60)
Female	9 (21)	52 (33)		4 (40)
Smoking history				
Never	14 (33)	8 (5)	<0.001	3 (30)
Former	21 (50)	128 (81)		1 (10)
Active smoker	7 (17)	22 (14)		5 (50)
Alcohol use				
Never	12 (28.6)	13 (8.2)	<0.001	4 (40)
Former	0 (0)	16 (10.1)		2 (20)
<2 units/day	10 (23.8)	18 (11.4)		1 (10)
2–6 units/day	17 (40.5)	76 (48.1)		2 (20)
>6 units/day	3 (7.1)	35 (22.2)		0 (0)
Overall AJCC stage				
Stage I–II	4 (9.5)	22 (14)	0.451	–
Stage III–IV	38 (90.5)	136 (86)		
AJCC tumor size^1^				
T1–2	21 (50)	52 (33)	0.047	–
T3–4	20 (48)	106 (67)		
AJCC nodal stage^2^				
N0	4 (9.5)	45 (28.5)	0.017	–
N1–3	38 (90.5)	110 (69.6)		
Treatment				
RT	13 (31)	54 (34)	0.776	–
RT/chemotherapy	19 (45)	74 (47)		
S/S + RT/S + RT + chemotherapy	10 (24)	30 (19)		
Second primary tumors				
Negative	41 (98)	138 (87)	0.084	–
Positive	1 (2)	20 (13)		

HPV, human papillomavirus; RT, radiotherapy; S, surgery; AJCC, American Joint Committee on cancer.

1 One missing;

2 three missing values.

### Methylation status by MS-MLPA

#### Cumulative methylation

**Figure 1:**
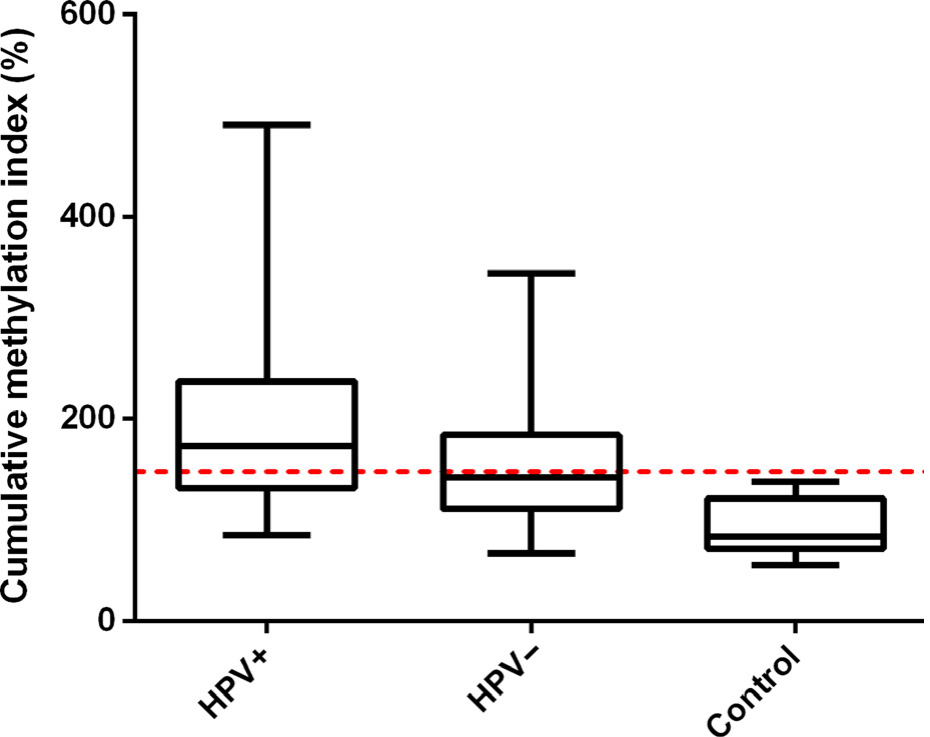
Cumulative methylation index of HPV-positive OPSCC (*n* = 42), HPV-negative OPSCC (*n* = 158), and control oropharynx tissue (*n* = 10) depicted in box plots. In box plots, the horizontal line in the middle of each box indicates the median; the top and bottom borders of the box mark 75th and 25th percentiles, respectively; and the whiskers above and below mark the range. Dashed line: cutoff value of 148.

The results of overall methylation (CMI) are shown in Figure1. Compared to control tissue (median = 84) we found a significantly higher CMI in HPV-positive tumors (median = 174, *P* < 0.001) and HPV-negative tumors (median = 142, *P* < 0.001). HPV-positive OPSCC showed a significantly higher (*P* = 0.008) CMI compared to HPV-negative OPSCC (mean CMI = 174 vs. 142, respectively). CMI was dichotomized using the median value (148) of the complete cohort of tumors. After multivariate logistic regression correcting for alcohol use, tumor size, lymph node status, and smoking, HPV presence was significantly associated with a high CMI (>148) (*P* = 0.004).

#### Gene related

**Figure 2:**
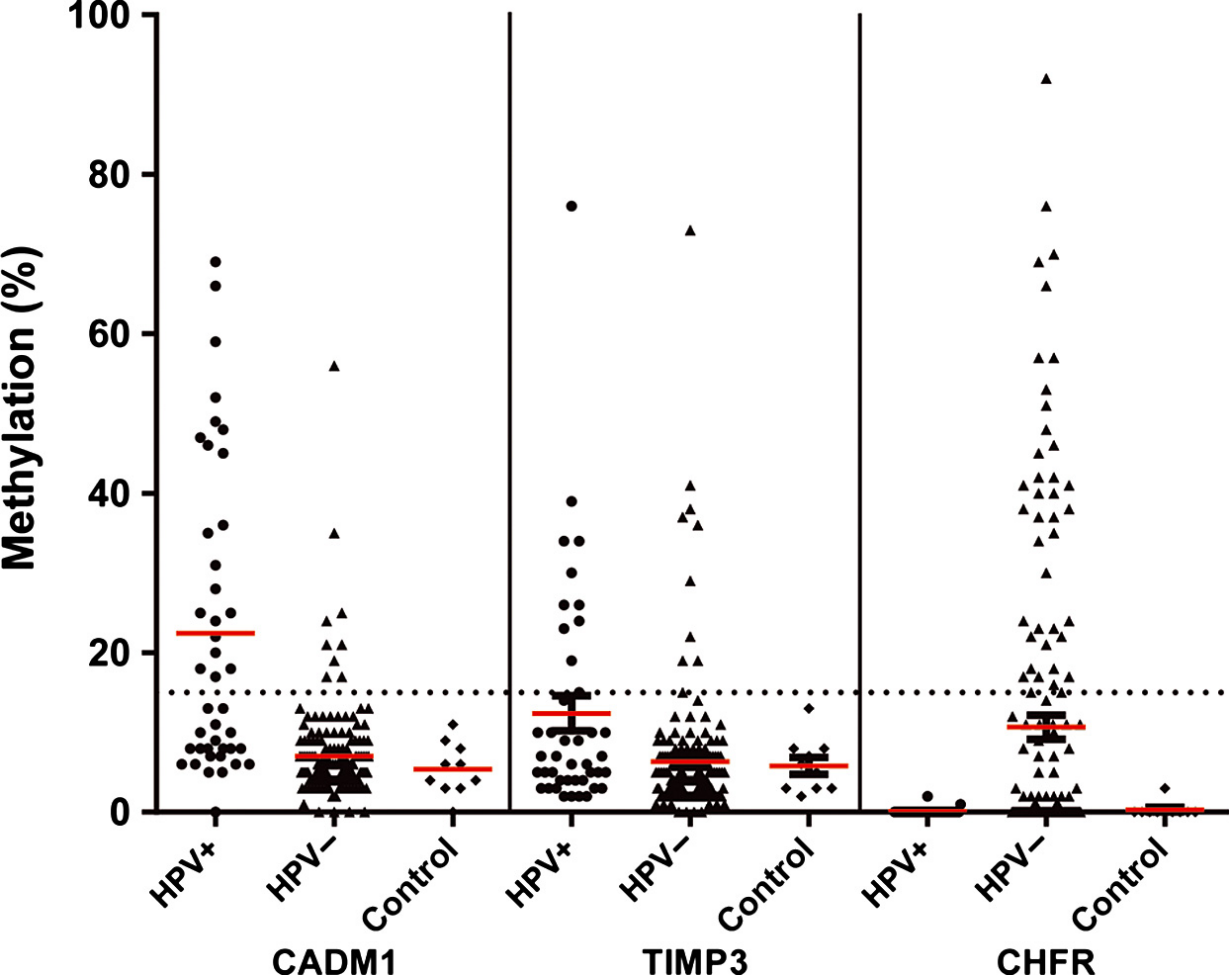
Extent of methylation of three HPV-related genes in HPV-negative OPSCC (*n* = 42, filled triangles), HPV-positive OPSCC (*n* = 158, filled circles), and control tissue (*n* = 10, filled diamonds) for each sample. Red line: mean value. Dashed line: cutoff value of 15% for promoter hypermethylation.

Table 2 shows the comparison of hypermethylation frequencies (number of samples with methylation value above cutoff value of 15%) of the 24 studied genes in HPV-positive and HPV-negative tumors. Normal oropharynx control samples showed no promoter hypermethylation in the studied genes, except for *CDH13* (40%), but the latter was still lower in control tissue (mean 13, range 7–22) compared to tumor tissue (HPV-positive: mean = 24, range 11–77; HPV-negative: mean = 20, range 6–96). No hypermethylation was observed for *BRCA1*, *BRCA2*, *HIC1*, *VHL*, *ATM*, *CD44*, *CDKN1B*, and *GST1P* in both HPV-positive and HPV-negative tumors. Hypermethylation frequencies for *CDH13*, *RARB*, and *DAPK1* were similar for HPV-positive and HPV-negative patients. Using logistic regression, thereby allowing for the controlling of confounders (alcohol use, tumor size, lymph node status, and smoking), patients positive for HPV16 had increased hypermethylated *CADM1* (OR = 19.3, CI = 7.5–49.3) or *TIMP3* (OR = 5.9, CI = 2.3–15.6). In contrast, an inverse correlation was found between HPV status and methylation of *CHFR* (OR = 0.1, CI = 0.013–0.80). (Fig.2).

**Table 2:** Genes present in the ME001-C2 tumor suppressor-1 MS-MLPA kit and frequencies of promoter hypermethylation (cutoff 15%) in 200 OPSCC patients divided by HPV status

Gene	Chromosome	Observed hypermethylation percentage (%)		*P*-value	Mean methylation, control tissue (%)

		HPV-positive	HPV-negative		
*CDH13*	16q23.3	86	65	0.014	Yes (13 range 4–22)
*DAPK1*	9q21.33	33	21	0.137	No (7 range 0–12)
*RARB*	3p24.2	40	34	0.512	No (7 range 4–14)
*TIMP3*	22q12.3	26	6	<0.001	No (6 range 2–13)
*CADM1*	11q23.3	50	6	<0.001	No (5 range 0–11)
*CHFR*	12q24.33	0	25	0.001	No (0 range 0–3)
*TP73*	1p36.32	19	13	0.487	No (7 range 2–13)
*APC*	5q22.2	12	9	0.577	No (3 range 2–6)
*ESR1*	6q25.1	10	7	0.524	No (7 range 4–10)
*CDKN2B*	9p31.3	5	3	0.608	No (2 range 0–4)
*CDKN2A*	9p21.3	5	1	0.112	No (4 range 0–10)
*FHIT*	3p14.2	5	1	0.195	No (2 range 0–5)
*RASSF1*^1^	3p21.31	0	3	0.581	No (2 range 1–4)
*MLH1*^1^	3p22.2	0	1	1	No (1 range 1–2)
*CASP8*	2q33.1	2	1	1	No (2 range 0–3)
*PTEN*	10q23.3	2	1	0.889	No (5 range 3–9)
*HIC1*	17p13.3	0	0	–	No (2 range 0–4)
*CDKN1B*	12p13.1	0	0	–	No (2 range 0–4)
*VHL*	3p25.3	0	0	–	No (1 range 0–2)
*CD44*	11p13	0	0	–	No (5 range 2–8)
*BRCA1*	17q21.31	0	0	–	No (1 range 1–2)
*ATM*	11q22.3	0	0	–	No (1 range 0–4)
*GSTP1*	11q13.2	0	0	–	No (4 range 3–9)
*BRCA2*	13q12.3	0	0	–	No (2 range 1–5)

The last column shows whether any methylation was present in normal oropharynx tissue (cutoff 15%, Yes or No) and shows the mean percentage of methylation and corresponding range.

1 For these genes, probes for two different CPG sites (a and b) are present in used MS-MLPA kit.

The methylation status of each gene was tested for an association with the clinical characteristics age, sex, tumor size (clinical T status), cervical lymph node status (N), distant metastases (M), clinical stage, alcohol, and nicotine consumption. Promoter hypermethylation status was not associated with lymph node status, sex, or tumor size for any of the genes. Hypermethylation of *CADM1* was associated with higher age (*P* = 0.050), no history of alcohol abuse (*P* = 0.007), or smoking (*P* < 0.001). Hypermethylation of *CDH13* was inversely related to distant metastasis (*P* = 0.022). Hypermethylation of *TIMP3* was more often present in patients with no history of smoking (*P* = 0.036). After correction for multiple comparisons, there was only significant correlation between promoter hypermethylation of *CADM1* and smoking.

### Comparison with squamous cell carcinoma of the cervix

**Figure 3:**
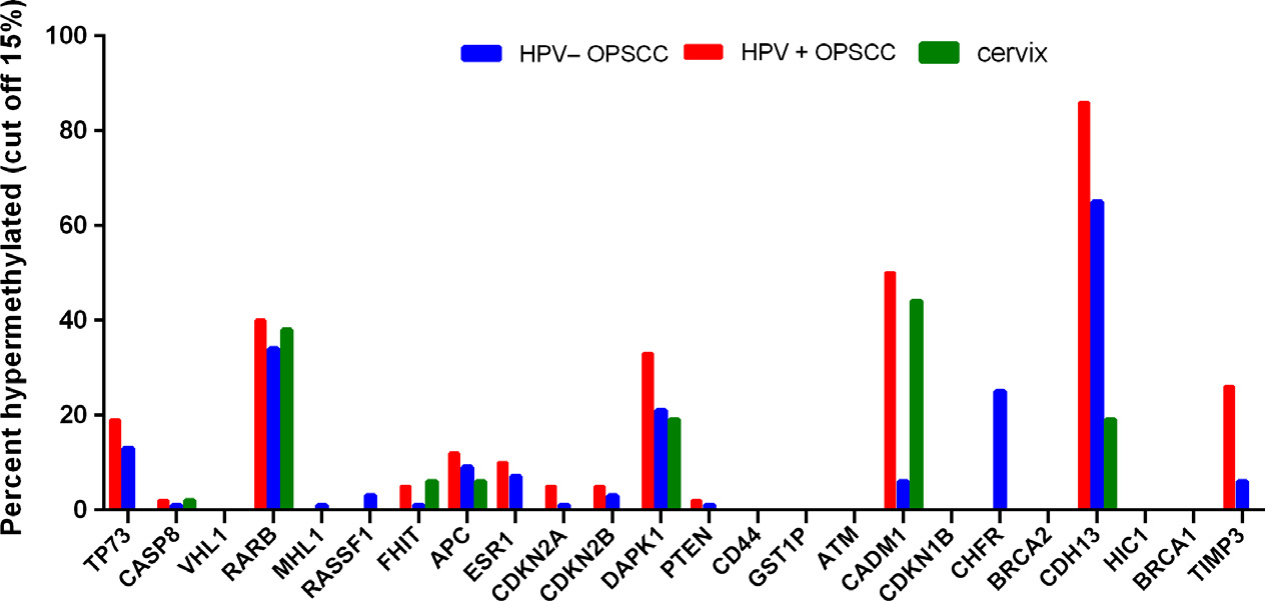
Promoter hypermethylation (>15% methylation) of 24 studied tumor suppressor genes in HPV-negative OPSCC (*n* = 158), HPV-positive OPSCC (*n* = 42), and cervical SCC (*n* = 16).

Methylation results could be obtained for 16/18 cervical SCC cases with clinical features as described in Table S2. Normal cervix control samples showed no promoter hypermethylation in the studied genes (Table S2). Figure3 shows hypermethylation frequencies of the 24 studied genes in HPV-positive OPSCCs (*n* = 42) in comparison with cervical SCCs (*n* = 16). Compared to cervical SCC, we observed a significantly higher CMI in HPV-positive OPSCCs (*P* = 0.037). The high frequencies of hypermethylation in *RARB* (40% vs. 38%), *CADM1* (50% vs. 44%), and *DAPK1* (33% vs. 19%) were shared between HPV-positive OPSCC and cervical cancer. Hypermethylation was however less frequent in cervical cancer in most genes, particularly in *CDH13* (*P* < 0.001) and *TIMP3* (*P* = 0.025). After correction for multiple comparisons, there was no significant correlation between promoter hypermethylation of *TIMP3* and tumor location (cervix vs. oropharynx). Univariate logistic regression analysis showed significant differences for alcohol, no other clinical feature was significantly different between the groups. After multivariate logistic regression analysis only methylation status of *CDH13* (OR = 66, CI = 6.4–678) was a strong independent predictor of location of HPV-related SCC.

### Survival

HPV positivity (*P* < 0.001) and small tumor size (T1–2) (*P* < 0.001) were correlated with increased 2 year overall survival as expected. Positive cervical lymph node status (N) (*P* < 0.001) and active smoking (*P* = 0.035) were associated with decreased overall survival. CMI dichotomized using a threshold of 148 was not significantly associated with patients' outcome. Patients with hypermethylation of *CADM1* (*P* = 0.004) had however increased survival compared to patients who did not have *CADM1* hypermethylation. Methylation status of no other individual gene was associated with patient survival. Combination score of HPV-related genes (*CADM1*, *TIMP3*, and inversed *CHFR*; scoring calculated: 1 point per methylated gene and in case of CHFR inversed with a total score ranging from 0 to 3) with a cutoff value of 2 or more showed a significant better overall survival (*P* = 0.018). In Cox regression, *CADM1* methylation, active smoking, HPV status, T status dichotomized, N status dichitomized, and the combined methylation pattern of HPV-related genes were included. *CADM1* methylation, active smoking, and the combined methylation pattern of HPV-related genes were not independent prognostic factors. In the subset of HPV-positive and HPV-negative tumors, methylation status of none of the individual genes was associated with patients' outcome.

### Correlation between CHFR and CADM1 protein level and promotor hypermethylation

To investigate the effect of promoter hypermethylation of *CHFR* and *CADM1* on transcriptional level, immunohistochemistry was used to compare *CHFR* and *CADM1* protein expression in hypermethylated OPSCC and OPSCC without hypermethylation.

For *CADM1*, reliable results were obtained for both protein expression and methylation status in 178 cases and for *CHFR* in 181 cases (15 of 193 for *CADM1* samples and 12 of 193 in *CHFR* samples were excluded from analyses because cores contained less than 5% tumor tissue or 95% contained no tissue and). *CADM1* expression was reduced in 89% (158/178) and *CHFR* expression in 47% of OPSCC. Using the cutoff value of 15% for promoter hypermethylation, the frequencies of *CADM1* promoter hypermethylation in this cohort was 16% (28/178) and of *CHFR* promoter hypermethylation was 18% (33/181). No correlation was found between percentages of methylation and protein expression for *CADM1* and *CHFR* as continuous variables (correlation coefficient = 0.052, *P* = 0.492; correlation coefficient = 0.111, *P* = 0.134), as well as categorical (*P* = 0.509; 0.335). Next, we investigated if *CADM1* and *CHFR* protein expression by IHC was related to HPV status. *CADM1* expression was not related to HPV status. Reduced protein expression of *CHFR* was significantly (*P* = 0.012) related to HPV-positive OPSCC in univariate analyses. To account for potential confounders of these results, we conducted a logistic regression analyses to adjust for baseline characteristics (age, gender, smoking, alcohol use, clinical T and N stage). After multivariate adjustment, reduced *CHFR* expression was an even more strongly related to HPV-positive OPSCC.

## Discussion

HPV-positive and HPV-negative OPSCC are driven by distinct carcinogenic pathways which are reflected in their diverse clinical behavior 1. These differences in underlying biology of HPV-positive and HPV-negative OPSCCs have been shown on genomic, transcriptomic, and protein expression levels 36,37. Our study further underscores this diversity by illustrating that HPV-positive and HPV-negative OPSCCs also differ epigenetically.

MS-MLPA of 24 common tumor suppressor genes resulted in the identification of three genes significantly differentially methylated in HPV-positive and HPV-negative OPSCC and three genes frequently methylated in both tumors. The genes *DAPK1*, *RARB*, and *CDH13* showed promoter hypermethylation in more than 20% of cases, both HPV-positive and HPV-negative tumors, indicating that these genes are often involved in HPV-positive and HPV-negative OPSCC carcinogenesis. These results are in accordance with previous studies evaluating promoter hypermethylation in HNSCC, although methylation rates vary widely in literature 26,38.

In addition, the frequency of promoter hypermethylation of *CDH13* was lower in cervical SCC compared to HPV-positive OPSCC, indicating that methylation of the promoter region of this gene is more strongly associated with oropharyngeal region. It has to be noted however that using a cutoff value of 15%, *CDH13* methylation was also a common event in control tissue, although at a lower frequency compared to OPSCC cases. In the literature there is no consensus on the threshold value for hypermethylation. Used cutoff values vary between studies ranging from 10% to 20% 19,27. Probably, for *CDH13*, the cutoff value to provide best discrimination between tumor and normal tissue (generally not methylated) should be higher. In addition, using a higher cutoff value of 22% (highest level of methylation in normal tissue) instead of 15%, hypermethylation of *CDH13* occurs still often in both HPV-positive and HPV-negative OPSCC (38 vs. 23%) and is not significantly different (*P* = 0.045) after Bonferroni correction.

Hypermethylation of genes in HNSCC in association with HPV status has been studied before. However, most of these studies used a small sample size, investigated a limited number of genes and in only four studies a cohort of tumors from exclusively oropharynx was evaluated in association with HPV status 15,16,21. To our knowledge, our study is the largest, most homogeneous cohort of OPSCC with known HPV status and overall survival described so far. In accordance with literature data, our study showed that promoter methylation (CMI) was more common in HPV-positive tumors compared to HPV-negative tumors 23. This could be explained by increased expression of DNA methyltransferases DNMT1 and DNMT3b due to HPV oncoproteins E6 and E7 39. In our study, the genes *TIMP3* and *CADM1* showed a significantly higher methylation frequency in HPV-positive tumors compared to HPV-negative tumors. In addition, an inverse correlation was found between HPV16 infection and methylation of *CHFR*.

*TIMP3* promoter methylation has been described in a wide range of tumors, including kidney, esophagus, colon, and breast cancer 40–43. *TIMP3* is a matrix metalloproteinase inhibitor and therefore able to inhibit growth, angiogenesis, invasion, and metastasis of tumors 43–45. Our data are consistent with a previous study from Weiss et al. 19 that described the correlation between HPV status and promoter methylation of *TIMP3* in a cohort of HNSCC, suggesting *TIMP3* is important for HPV-positive OPSCC carcinogenesis. However, in cervical cancer no promoter hypermethylation of this gene was observed in our study, suggesting that methylation of this gene might be location specific.

Our data show for the first time a linkage between HPV16 positivity and *CADM1* methylation in OPSCC. *CADM1* plays a role in cell–cell adhesion and reduced expression of this gene is correlated with lymph node metastasis 46. Interestingly, *CADM1* was also often methylated in cervical cancer (Fig.2) and reduced expression of *CADM1* has been reported in cervix squamous cell carcinomas 27. This confirms that promoter methylation of *CADM1* might be induced by HPV16. Promoter methylation of the *CADM1* gene has also been described in several other types of cancer, including nonsmall cell lung carcinoma, pancreatic cancers, and cervical carcinomas 27,47.

Another finding in this study is that the promoter region of *CHFR* was not methylated in any of the HPV-positive OPSCC or cervical SCC cases. *CHFR* is a gene involved in a checkpoint regulating entry to mitosis 48. So far, methylation of *CHFR* has not been analyzed in OPSCC in association with HPV status. The methylation frequency of the *CHFR* gene in HPV-negative tumors is in concordance with previous data reported for HNSCC, although the results in HPV-positive OPSCCs and cervical SCC are in contrast to a previous publication showing *CHFR* promoter methylation in HPV-positive cervix carcinomas 27, 38. Inconsistent results in literature may be explained by differences in used methylation assays, type of tested material, composition of the cohorts, and the use of different cutoff values for hypermethylation. Since HPV-negative OPSCCs are associated with tobacco and alcohol use, a correlation of methylation status with these variables appears to be plausible. However, in our study no correlation was found between smoking or alcohol use and methylation status of *CHFR*, suggesting that promoter methylation of this gene is induced by another mechanism.

The correlation between promoter hypermethylation and subsequent mRNA or protein expression is notorious and gene dependent 49, 50. Indeed, we found that *CADM1* and *CHFR* promoter hypermethylation poorly correlates with protein expression. *CADM1* protein expression was reduced in 89% of the oropharyngeal tumors, suggesting that next to epigenetic silencing other nonepigenetically mechanism might account for the reduced expression. Besides, Overmeer et al. 51 showed in cervical tumors that only dense methylation (>methylated two regions) of *CADM1* is associated with reduced protein expression. In addition, a strong correlation was found between HPV positivity and reduced expression of the *CHFR* protein, while no promoter methylation of *CHFR* was observed in HPV-positive group. In contrast, the protein expression of *CHFR* was high in HPV-negative patients, whereas in HPV-negative patients 25% of this gene was hypermethylated. This might be explained by posttranscriptional and translational alterations and crosstalk. 52.

Interestingly, the following three genes, *RASSF1A*, *MLH1*, and *CDKN2A*, have been identified to be methylated in HNSCC in previous studies, but were only rarely methylated in our cohort. Methylation studies in HNSCC have been extensively described over the past years, however, wide ranges have been reported in these methylation data 26. The heterogeneity of these results could be explained by differences in composition of cohort, differences in used methods for methylation analyses and tumor specimens. Many studies used methylation-specific PCR with bisulfite-modified templates for detection of gene methylation, which is simple and affordable but not quantitative and prone to overestimation of methylation due to incomplete bisulfite conversion as shown by Yalniz et al. for *RASSF1* 38,53. In addition, different thresholds were used as discussed before and most studies evaluated methylation in HNSCC overall, not per anatomical sublocation. Another explanation could have been a too low tumor cell percentage. However, this is unlikely as in our cohort the tumor percentage was minimally 70% in 80% of the samples. In addition, literature shows that MS-MLPA can also be used in samples with mixed populations of cells – as long as 30% of methylated DNA/tumor DNA is present in the sample, the methylation status will be recognized correctly 54. All these parameters can contribute to the large variety in methylation results in literature.

This study has several limitations. MS-MLPA cannot distinguish between partially and fully methylated promoter regions, since this method measures methylation for only one CpG site in promoter region. However, for gene silencing not all CpG islands in promoter region have to be methylated 28,54. In addition, multiple studies showed that MS-MLPA and quantitative multiplex methylation-specific PCR (QM-MSP) showed a good correlation, suggesting that MS-MLPA is good and reliable method to detect methylation 55,56. While the sample size of oropharyngeal cancer was rather high, this is not the case for the normal tissue samples. This can be explained by the limited number of samples available, as they had to be matched for age, alcohol use, and smoking, because these factors can also induce methylation. Kostareli et al. 16 showed a HPV-related promoter methylation pattern of five genes with a strong correlation with patients' outcome in OPSCC. In our study, univariate analysis of *CADM1* promoter methylation was associated with a better overall survival. However, after correction for confounders in a multivariate model there was no individual methylated gene, neither overall methylation level (CMI) nor any combination of HPV-related genes that could independently predict overall survival, in the entire cohort or in the subset of only HPV-positive tumors. This may be explained by the fact that in multivariate analysis, HPV status is the strongest independent predictor for survival and promoter methylation of *CADM1* is significantly associated with HPV status.

In conclusion, our findings suggest that promoter methylation is important in the development of OPSCC, in both HPV-positive and HPV-negative tumors. Promoter methylation was more common in HPV-positive compared to HPV-negative OPSCC. Hypermethylation of the genes *CADM1*, *TIMP3*, and *CHFR* was significantly different between HPV-positive and HPV-negative tumors. However, this is not reflected on protein level suggesting that other (posttranslational or transcriptional) mechanisms regulate the expression of these proteins.

## Supporting Information

**Table S1.** Genes present in the ME001-C2 MS-MLPA kit. Probe length, chromosomal position, function, and hallmark are described for each of the 24 genes.

**Table S2.** Genes present in the ME001-C2 tumor suppressor-1 MS-MLPA kit and frequencies of promoter hypermethylation (cutoff 15%) in cervical squamous cell carcinoma.
