# Supplementary material for: HPV-positive oropharyngeal squamous cell carcinoma is associated with TIMP3 and CADM1 promoter hypermethylation
Source: Cancer Med. 2014 Jul 26;3(5):1185–96. doi: 10.1002/cam4.313 (PMC4302669; doi:10.1002/cam4.313)
Supplement: Supplementary file 1 — Table S1. Genes present in the ME001-C2 MS-MLPA kit. Probe length, chromosomal position, function, and hallmark are described for each of the 24 genes. [file cam40003-1185-SD1.doc]

| **TABLE S1.** Genes present in the ME001- C2 MS-MLPA kit. Probe length, chromosomal position, function and hallmark are described for each of the 24 genes. | | | | | |
| --- | --- | --- | --- | --- | --- |
| **Length** | **Gene** | **Chromosome** | **Function** | **Hallmark(50)** | **Mapview** |
| 142 | *TIMP3* | 22q12.3 | Inhibition of tumor growth and angiogenesis | Activating invasion and metastasis | 22-031,527795 |
| 148 | *APC* | 5q22.2 | Wnt-pathway antagonist | Activating invasion and metastasis | 05-112,101357 |
| 161 | *CDKN2A* | 9p21.3 | Cell cycle-cyclin kinase inhibitor | Evading growth suppressors | 09-021,985276 |
| 167 | *MLH1A** | 3p22.2 | DNA-repair | Genome instability and mutation | 03-037,010000 |
| 184 | *ATM* | 11q22.3 | DNA damage sensor | Genome instability and mutation | 11-107,599044 |
| 193 | *RARB* | 3p24.2 | Inhibits cell growth | Evading growth suppressors | 03-025,444559 |
| 211 | *CDKN2B* | 9p31.3 | cell growth regulator | Evading growth suppressors | 09-021,998808 |
| 220 | *HIC1* | 17p13.3 | cell growth regulator | Evading growth suppressors | 17-001,905107 |
| 238 | *CHFR* | 12q24.33 | Early G2/M checkpoint | Sustaining proliferative signaling | 12-131,974372 |
| 246 | *BRCA1* | 17q21.31 | DNA repair | Genome instability and mutation | 17-038,530811 |
| 265 | *CASP8* | 2q33.1 | Pro-apoptosis | Resisting cell death | 02-201,830871 |
| 274 | *CDKN1B* | 12p13.1 | Cell cycle-cyclin kinase inhibitor | Evading growth suppressors | 12-012,761863 |
| 292 | *PTEN* | 10q23.3 | Negatively regulating AKT/PKB signaling pathway | Evading growth suppressors | 10-089,612348 |
| 301 | *BRCA2* | 13q12.3 | DNA repair | Genome instability and mutation | 13-031,787722 |
| 319 | *CD44* | 11p13 | Cell-cell interactions, cell adhesion and migration | Activating invasion and metastasis | 11-035,117389 |
| 328 | *RASSF1A** | 3p21.31 | RAS-pathway regulation | Sustaining proliferative signaling | 03-050,353347 |
| 346 | *DAPK1* | 9q21.33 | Pro-apoptosis | Resisting cell death | 09-089,303075 |
| 353 | *VHL* | 3p25.3 | Ubiquitination and degradation of hypoxia-inducible-factor | Inducing angiogensis | 03-010,158426 |
| 373 | *ESR1* | 6q25.1 | Cellular proliferation and differentiation | Sustaining proliferative signaling | 06-152,170883 |
| 382 | *RASSF1B** | 3p21.31 | RAS-pathway regulation | Sustaining proliferative signaling | 03-050,353298 |
| 400 | *TP73* | 1p36.32 | Angiogenesis and apoptosis | Activating invasion and metastasis | 01-003,558977 |
| 409 | *FHIT* | 3p14.2 | Mostly unknown, but associated with malignancy | Unknown | 03-061,211918 |
| 427 | *CADM1* | 11q23.3 | Cell- cell adhesion | Activating invasion and metastasis | 11-114,880585 |
| 436 | *CDH13* | 16q23.3 | Cell-cell adhesion | Activating invasion and metastasis | 16-081,218219 |
| 454 | *GSTP1* | 11q13.2 | Detoxification | Genome instability and mutation | 11-067,107774 |
| 463 | *MLH1B** | 3p22.2 | DNA-repair | Genome instability and mutation | 03-037,009621 |
| *For these genes, probes for two different promoter CpG sites are present. | | | | | |
