# Supplementary material for: HPV-positive oropharyngeal squamous cell carcinoma is associated with TIMP3 and CADM1 promoter hypermethylation
Source: Cancer Med. 2014 Jul 26;3(5):1185–96. doi: 10.1002/cam4.313 (PMC4302669; doi:10.1002/cam4.313)
Supplement: Supplementary file 2 — Table S2. Genes present in the ME001-C2 tumor suppressor-1 MS-MLPA kit and frequencies of promoter hypermethylation (cutoff 15%) in cervical squamous cell carcinoma. [file cam40003-1185-SD2.doc]

| **TABLE S2.** Genes present in the ME001-C2 tumour suppressor-1 MS-MLPA kit and frequencies of promoter hypermethylation (cutt-of 15%) in cervical squamous cell carcinoma | | | |
| --- | --- | --- | --- |
| **Gene** | **Chromosome** | **Observed hypermethylation percentage (%)** | **Hypermethylation normal tissue (range%)** |
| *CDH13* | 16q23.3 | 19 | No( 7-14) |
| *DAPK1* | 9q21.33 | 19 | No ( 0-5 ) |
| *RARB* | 3p24.2 | 38 | No ( 5-10) |
| *TIMP3* | 22q12.3 | 0 | No ( 2-4) |
| *CADM1* | 11q23.3 | 44 | No ( 3-5) |
| *CHFR* | 12q24.33 | 0 | No (0 ) |
| *TP73* | 1p36.32 | 0 | No (6-12) |
| *APC* | 5q22.2 | 13 | No (3-5) |
| *ESR1* | 6q25.1 | 0 | No (0-5) |
| *CDKN2B* | 9p31.3 | 0 | No (2-6) |
| *CDKN2A* | 9p21.3 | 0 | No(3-6) |
| *FHIT* | 3p14.2 | 6 | No (0) |
| *RASSF1* | 3p21.31 | 0 | No (0-4) |
| *MLH1* | 3p22.2 | 0 | No (0-6) |
| *CASP8* | 2q33.1 | 6 | No (2-4) |
| *PTEN* | 10q23.3 | 0 | No (3-7) |
| *HIC1* | 17p13.3 | 0 | No (2-5) |
| *CDKN1B* | 12p13.1 | 0 | No (2-4) |
| *VHL* | 3p25.3 | 0 | No (0-2) |
| *CD44* | 11p13 | 0 | No (0-6) |
| *BRCA1* | 17q21.31 | 0 | No (1-3) |
| *ATM* | 11q22.3 | 0 | No (1-4) |
| *GSTP1* | 11q13.2 | 0 | No (4-7) |
| *BRCA2* | 13q12.3 | 0 | No (1-3) |
| The last column shows whether any methylation was present in normal cervical tissue (cut-off 15%, Yes or No) and shows the range of methylation. | | | |
